# A Mathematical Methodology for Determining the Temporal Order of Pathway Alterations Arising during Gliomagenesis

**DOI:** 10.1371/journal.pcbi.1002337

**Published:** 2012-01-05

**Authors:** Yu-Kang Cheng, Rameen Beroukhim, Ross L. Levine, Ingo K. Mellinghoff, Eric C. Holland, Franziska Michor

**Affiliations:** 1 Department of Biostatistics and Computational Biology, Dana-Farber Cancer Institute, and Department of Biostatistics, Harvard School of Public Health, Boston, Massachusetts, United States of America; 2 Cancer Biology and Genetics Program, Brain Tumor Center, Memorial Sloan-Kettering Cancer Center, New York, New York, United States of America; 3 Tri-Institutional Training Program in Computational Biology and Medicine, Weill Cornell Medical College, New York, New York, United States of America; 4 Departments of Cancer Biology and Medical Oncology, Dana-Farber Cancer Institute, Boston, Massachusetts, United States of America, Department of Medicine, Harvard Medical School, Boston, Massachusetts, United States of America, Department of Medicine, Brigham and Women's Hospital, Brigham and Women's Hospital, Boston, Massachusetts, United States of America, and Cancer Program, The Broad Institute of MIT and Harvard, Cambridge, Massachusetts, United States of America; 5 Human Oncology and Pathogenesis Program, Memorial Sloan-Kettering Cancer Center, New York, New York, United States of America; ETH Zürich, Switzerland

## Abstract

Human cancer is caused by the accumulation of genetic alterations in cells. Of special importance are changes that occur early during malignant transformation because they may result in oncogene addiction and thus represent promising targets for therapeutic intervention. We have previously described a computational approach, called Retracing the Evolutionary Steps in Cancer (RESIC), to determine the temporal sequence of genetic alterations during tumorigenesis from cross-sectional genomic data of tumors at their fully transformed stage. Since alterations within a set of genes belonging to a particular signaling pathway may have similar or equivalent effects, we applied a pathway-based systems biology approach to the RESIC methodology. This method was used to determine whether alterations of specific pathways develop early or late during malignant transformation. When applied to primary glioblastoma (GBM) copy number data from The Cancer Genome Atlas (TCGA) project, RESIC identified a temporal order of pathway alterations consistent with the order of events in secondary GBMs. We then further subdivided the samples into the four main GBM subtypes and determined the relative contributions of each subtype to the overall results: we found that the overall ordering applied for the proneural subtype but differed for mesenchymal samples. The temporal sequence of events could not be identified for neural and classical subtypes, possibly due to a limited number of samples. Moreover, for samples of the proneural subtype, we detected two distinct temporal sequences of events: (i) RAS pathway activation was followed by TP53 inactivation and finally PI3K2 activation, and (ii) RAS activation preceded only AKT activation. This extension of the RESIC methodology provides an evolutionary mathematical approach to identify the temporal sequence of pathway changes driving tumorigenesis and may be useful in guiding the understanding of signaling rearrangements in cancer development.

## Author Summary

Cancer is a deadly disease that develops through the accumulation of genetic changes over time. Many biological models do not incorporate this temporal aspect of tumor formation and progression, in part due to the difficulty of determining the sequence of events through biological experimentation for most cancer types. We previously developed a computational algorithm with which we can quickly and cost-effectively determine the order in which mutations arise in the tumor even when large numbers of mutations are considered. In this paper, we extended our method to incorporate biological knowledge of the common pathways by which cancer progresses. We applied these techniques to primary glioblastoma, the most common form of brain cancer. We found that when all samples are taken into account, a temporal sequence of pathway events emerges; however, different subtypes of glioblastoma vary in their temporal sequence of events. This algorithm can also be easily applied to other cancer types as clinical data becomes available, showing the benefit of computational and mathematical tools in cancer research. Using temporal information, cancer biologists will be able to develop more accurate animal models of tumor formation and learn more about how mutations interact in time, thus leading to better treatments for cancer.

## Introduction

New high-throughput sequencing and microarray technologies provide researchers with access to increasingly large and complex datasets comprising genome-level alterations in cancer [1], [2], [3]. Computational algorithms have been designed to sift through this data with the goal of uncovering mutational patterns that are typical for a particular cancer type and consistent between sample sets [1], [2], [4], [5]. However, the ability to functionally validate recurrent genetic events in transgenic mouse models and human cell lines is limited by the lack of knowledge of the temporal order in which these alterations arise during tumorigenesis. The temporal sequence of events is important since it can inform the correct genomic background in which a mutation must arise to confer an oncogenic phenotype to cells. Furthermore, it may contribute to drug discovery since genomic alterations arising early during tumorigenesis may be more likely to induce oncogenic addiction and may thus represent promising therapeutic targets [6]. In some cancers, such as colorectal cancer, the order of events can be determined through the analysis of several pre-malignant stages [7], [8]. Most cancer types, however, do not present with clinically observable precursor stages, and therefore the identification of the temporal sequence of events using biological or clinical approaches is difficult.

There is a growing literature of mathematical, statistical and computational approaches to determining the temporal sequence of events arising during tumorigenesis. Previously published methods include the linear model [7], the oncogenetic tree (oncotree) approach [9], [10], [11], [12], various Bayesian graphical approaches [13], [14], and some clustering-based methods [15], [16]. Based upon the seminal work in delineating the temporal sequence of events in colorectal cancer by Vogelstein and colleagues, the linear model assumes that there exists a single, most likely order of mutations, and that all of these mutations arise in sequential order [7]. The oncogenetic tree approach generalizes the assumption of a single sequential path by providing a tree structure to the temporal sequence of mutations, allowing for diverging temporal orderings of events [9], [10]. In probabilistic oncotrees, the tree structure represents the probabilities of accumulating further mutations along divergent temporal sequences [9]. An alternative distance-based oncotree approach involves generating a phylogenetic tree over all events using a distance measure between mutational events, where leaf nodes represent the set of possible events. The closer a leaf node is to the root, the earlier the corresponding mutation arises [10]. Further development of the probabilistic oncotree methodology by Beerenwinkel et al. resulted in the mixture tree model, in which multiple oncogenetic trees, each of which can result in cancer development independently, are included in the model [12]. The consideration of multiple tree structures allows for inclusion of multiple independent temporal sequences of events that can result in the development of cancer. Notably, one tree structure that the mixture tree model includes is a star-shaped tree predicting that every mutation arises independently, accounting for random mutations that arise but are not involved in any temporal sequence of events. An expectation maximization algorithm is then used to determine the most likely tree mixture to fit the data [12]. This approach has often been used to analyze CGH data [17], [18], [19], [20], [21], [22], [23]. However, one acknowledged restriction of tree-based methods is that the tree structure precludes the possibility of converging evolutionary paths [13] that occur when multiple alterations result in the same phenotypic effect. Furthermore, tree-based models impose a strict ordering of events: if an event occurs in a leaf of the tree, then it necessarily must be preceded by all events between the leaf and the root of the tree. Bayesian graphical methods, by allowing any network structure, can include converging evolutionary paths [13], [14], however at the cost of additional computation necessary to search the expanded multi-dimensional result space.

**Figure 1:**
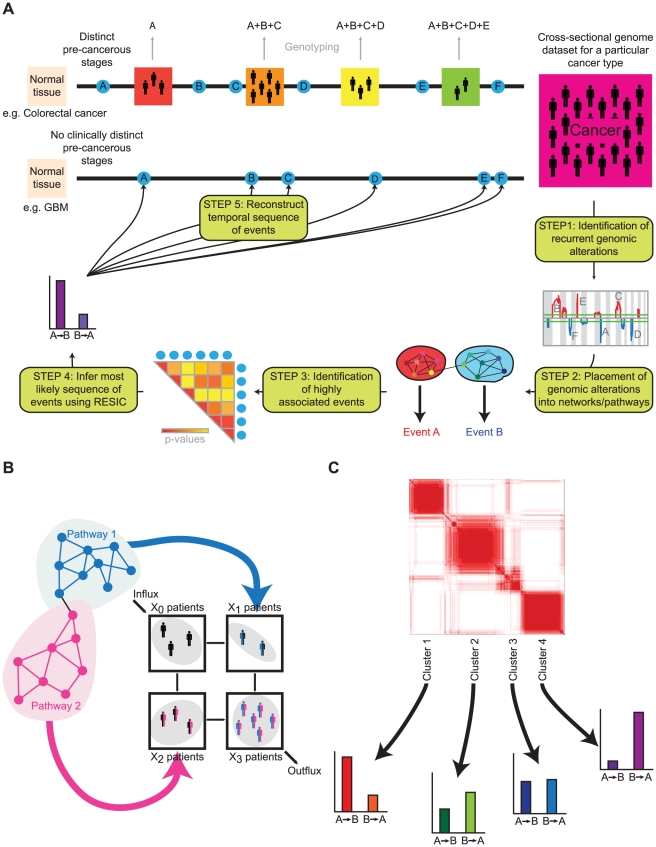
The methodology of pathway-driven RESIC. **A**) A schematic diagram of pathway-driven RESIC. For those cancer types for which clinico-pathologically defined stages can be identified, such as colorectal cancer, the temporal sequence in which genetic alterations arise during tumorigenesis can be inferred through genotyping of samples from patients at different stages of disease progression. We previously designed an evolutionary computational algorithm called *R*etracing the *E*volutionary *S*teps *I*n *C*ancer (RESIC) to determine the temporal order of somatic mutations for cancer types that are diagnosed *de novo* without detectable precursor lesions (e.g. primary glioblastoma) through the use of genomic data from a large number of samples (one per patient) of a particular histological type [24]. We extended this methodology to analyze the temporal sequence of functional alterations in signaling pathways. We begin with a genomic dataset of patient samples classified as the tumor (sub)type of interest. As Step 1, we use an algorithm such as GISTIC [5] to identify recurrent genetic aberrations in the dataset. In Step 2, we combine genetic alterations identified as impacting specific signaling pathways into single alteration events. In Step 3, we identify statistically significantly correlated events that occur sufficiently frequently. In Step 4, the most likely sequence for each set of associated events is identified using RESIC. The results generated from the RESIC analyses are used to reconstruct the order in which pathway alteration events arise during the development of a particular cancer type (Step 5). Our methodology is applicable to large-scale datasets and can be used to identify the temporal sequence of pathway alterations in cancer. **B**) Functional modules in signaling result in single events analyzed in RESIC. **C**) Gene expression-based subtypes can be split up into separate RESIC analyses or analyzed as a combined dataset.

We have previously described a computational approach, called *R*etracing the *E*volutionary *S*teps *I*n *C*ancer (RESIC) [24], which determines the temporal sequence of specific genetic events for primary tumor types for which cross-sectional genomic data is available (Figure 1A). This approach can be used to resolve the relative order of genetic events with respect to other alterations of interest; however, in the absence of further data, the time of emergence of these events relative to phenotypes such as malignancy or metastasis cannot be identified. In the RESIC model, we adopted a different approach as compared to prior work: RESIC explicitly considers the evolutionary dynamics of mutation accumulation within a population of patients. Each patient harbors a collection of self-renewing cells that are at risk of accumulating the alterations leading to cancer; this cell population follows a stochastic process known as the Moran model [25]. This stochastic process model of mutation accumulation was then approximated with a dynamical systems model whose steady state distribution across all possible mutational states can be compared with the frequencies of patients harboring the corresponding genetic events. The resulting fitness values conferred by genomic alterations, obtained by an optimization algorithm to minimize the distance between the observed and predicted patient frequencies, were then used to determine the relative order of events arising in a patient population [24]. RESIC analyses are performed for sets of correlated genetic events, thus resulting in a relative ordering of alterations. The use of pair-wise comparisons also allows for converging temporal orderings. A detailed explanation of the RESIC algorithm is provided in the Methods section.

In many cases, specific genetic alterations are not necessary for malignant transformation; instead, particular oncogenic phenotypes must be achieved through the emergence of any of a number of alternative mutations (Figure 1B) [26], [27], [28], [29], [30]. In addition, many cancers can be subdivided into distinct molecular subtypes (Figure 1C), which often result from differences in the set of genetic alterations accumulated and potentially the order in which they arise. Including pathway and subtyping information may alter the order in which genetic events arise during cancer progression. To address these issues, we have extended our RESIC methodology to consider both pathway-based phenotypic changes and the subtype-driven context of cancer in order to examine how the temporal sequence of events differs when such information is included. These two topics are considered in the analysis of primary glioblastoma (GBM). Due to its aggressiveness and poor prognosis, as well as its frequency, patient samples of GBM have been extensively collected and the data made easily accessible to researchers. In particular, The Cancer Genome Atlas (TCGA) project has provided measurements of multiple types of genomic alterations, with each sample processed in a uniform manner, for a large set of patient samples of GBMs [3]. The TCGA dataset provides an opportunity to effectively analyze the temporal sequence of pathway alterations using RESIC through an investigation of the specific signaling disruptions common to GBMs [31], [32], its well-defined molecular subtypes [33], [34], [35], and the availability of similarly treated patient samples [3]. We chose the RESIC methodology instead of previous approaches to investigate these issues since RESIC provides, in addition to the temporal ordering of events, the relative fitness values of cells harboring individual combinations of mutations. Furthermore, RESIC is capable of determining the order of different types of events, such as point mutations, focal amplifications and deletions, as well as whole chromosome and chromosome arm changes. Finally, RESIC is computationally efficient.

## Results

### Data and parameters

We gathered copy number alteration (CNA) data from 462 GBM patient samples through the TCGA Data portal at http://tcga-data.nci.nih.gov [3]. We then applied the GISTIC algorithm [5] to the segmented CNA data in order to determine the significance of all copy number alterations. In particular, we applied our method to focal amplifications and deletions, not whole chromosome alterations, as the former alterations are more likely to result in the alteration of only a single gene or a few genes. A subset of the patient samples (431) also included gene expression data, which we used for subtyping. Roughly a third of the samples (145) also included sequencing information [3], which we used to investigate the robustness of the results obtained when using solely CNA data. For this validation, we limited point mutations of interest to non-synonymous point mutations and small insertions or deletions (indels). For all RESIC analyses, we considered significant positive correlations between events using one-sided Fisher's Exact Test with a p-value cutoff of 0.05, as required for data pre-processing within our RESIC methodology in order to ensure co-occurrence of events. If mutational events do not co-occur sufficiently frequently, then the question about the temporal sequence of events is meaningless since the events may be associated with separate subtypes [24]. We used an uncorrected p-value cutoff of 0.05 instead of applying a correction for multiple testing in order to broaden the set of mutations to be included in our analyses. We also limited considered interactions to those pairs of genes with a marginal and joint frequency of alteration greater than 5%. Since each CNA may span several genes, the interpretation of results needs to take the identity of those genes and likely functional effects into account; a straight-forward interpretation of the results is to assign the obtained order to CNA events rather than individual genes. Since the genes within one CNA arose due to a common mechanism and are thus not independent, no conclusion can be drawn about the causal implications of each gene within the CAN for its emergence.

RESIC relies upon the transition rates between mutational states, which are a function of the number of cells at risk of accumulating the alterations (the population size), the rate at which the alterations arise (the mutation rate), and the change in growth rate that alterations confer to cells (the fitness) [24]. In the RESIC implementation, the optimization algorithm is used to identify the fitness values that minimize the distance between model prediction and patient data while keeping the population size and mutation rate estimates constant [24]. This choice was made due to the robustness of the results to changes in these latter parameters [24]. We calculated the rate at which focal CNAs arise per cell division by using the copy number alteration rate per locus per generation as determined in sperm, multiplied by the number of cell divisions during spermatogenesis (see Methods). Using those estimates, we obtained a CNA rate per locus per cell division of 2.2×10^−7^. This rate agrees with argumentation that point mutation and focal copy number alteration rates must be similar in scale in order for both types of alterations to be observed with similar frequency [36]. We used *μ* = 1.0×10^−7^ as the rate at which CNAs arise per allele per cell division, and *N* = 100 as the population size for computational speed; while the latter number may not accurately capture the order of magnitude of the number of stem cells per tissue compartment from which the tumor initiates, or the number of cancer stem cells per tumor, we have previously shown that the results of RESIC are not significantly affected by changes in the population size by orders of magnitude [24]. This robustness arises due to the scaling of the transition rates in the Markov process by the magnitude of the population size and holds as long as the system parameters are within a regime such that step-by-step evolution applies (i.e. one mutation arises and is lost or fixed before the next one emerges, [24]).

### RESIC analysis of CNA data provides similar results to analysis of point mutation and CNA data

To ensure the validity of our analysis using solely CNA data, we performed separate RESIC analyses of the CNA data alone; these results were then compared to those obtained using CNA and point mutation data in the subset of the samples for which both CNA and point mutation information was available. Similarly to our prior validation analyses [24], we expected that almost all orderings determined using CNA information only would either remain the same or weaken in significance. Indeed, we found that our results varied little when including point mutation data also. The only exception involved *TP53*, a gene known to be primarily altered through point mutations [37]. In the largest set of cases, 23/40 at the paired-gene level and 3/6 at the pathway level, the resultant orderings remained within 3% of each other while the dominant order stayed constant. In a second set of orderings, the findings decreased in significance when only CNA data was included (13/40 when analyzed using pairs of genes and 1/6 at the pathway level). The smallest group involved those orderings that differed between analyses using CNA data only and analyses using CNA plus point mutations, with 4/40 using gene-pair comparisons and 2/6 using pathway analyses. In this group, most orderings (5/6) involved *TP53*. The remaining ordering involved the PDGFRA locus, with point mutations occurring in amplified PDGFRA. RESIC analyses involving three-gene mutation diagrams were not significant due to the smaller set of samples available compounded with the larger number of samples required for three-gene analysis. Thus, with the exception of genes most commonly altered by point mutations, the orders determined using CNA information only are equivalent or weaker than those using point mutation data and CNA data. As such, use of CNA data alone at most provides weaker temporal sequences and at best provides equivalent temporal sequences as compared to analyses including point mutations also, unless a gene is known to be primarily altered via point mutations.

### A pathway-based RESIC analysis of GBM determines a consistent overall order of events

In many cancer types, a variety of genetic and/or epigenetic alterations result in a common phenotype required for cancer initiation and progression. In an effort to categorize the effects of specific genetic alterations, we classified these changes into the signaling pathways to which the altered genes belong. This choice was made since commonly mutated genes that reside in the same signaling pathway are expected to affect the progression of cancer in a similar manner; indeed, such commonality in phenotypic effects has been observed in multiple pathways involved in GBM [31], [38], [39], [40], [41], [42]. Therefore, any alteration in the set of genes within a single signaling pathway is expected to result in the same (or very similar) effects onto the reproductive success of cells carrying such alterations. We thus defined a pathway alteration as an event in which at least one of the genes involved in the pathway is genetically altered; for our purposes, pathways are defined following the approach of the TCGA project [3], [31], [32] (see Methods for details and a discussion of alternative approaches for pathway definition). Through the combination of mutational events into pathways, we are then able to obtain a broad view of the order of events in cancers in which conflicting signals obscure the order of events at the single gene level. We considered the TP53, PI3KC1/AKT, PI3KC2, RAS, and RB signaling pathways in GBM (Table 1). Inactivation of the TP53 pathway was defined by amplification of MDM2/4 or deletion of TP53 or CDKN2A; since sequence information was not available for most samples, point mutations inactivating TP53 could not be included. Activation of the PI3KC1/AKT pathway was defined by amplification of AKT1/2/3, GAB1, IRS1, PIK3CA/B/D/G, PIK3R1/2, PDPK1, or SRC, or deletion of PTEN. Activation of the PIK3C2 pathway referred to amplification of PIK3C2A/B/G. RAS pathway activation was defined by amplification of ARAF/BRAF/HRAS/KRAS/NRAS, EGFR, ERBB2/3, FGFR1/2, GRB2, IGFR, MET, PDGFRA/B, or RAF1, or deletion of CBL, ERRFI1, or SPRY2. RB pathway inactivation referred to amplification of CCND1/2, CCNE1, or CDK2/4/6, or deletion of CDKN1A/B, CDKN2A/B/C, or RB1 (Table 1). These pathways were defined as previously described using the Pathway Commons approach to investigate TCGA GBM data [31], [32].

**Table 1:** The definition of pathways.

*Positive Effects*				
TP53	PIK3C1/AKT	PI3KC2	RAS	RB
CDKN2A	AKT1	PIK3C2A	ARAF	CDKN1A
TTP53	AKT2	PIK3C2B	BRAF	CDKN1B
	AKT3	PIK3C2G	EGFR	CDKN2A
	GAB1		ERBB2	CDKN2B
	IRS1		ERBB3	CDKN2C
	PIK3CA		FGFR1	RB1
	PIK3CB		FGFR2	
	PIK3CD		GRB2	
	PIK3CG		HRAS	
	PIK3CR1		IGFR	
	PIK3CR2		KRAS	
	PDPK1		MET	
	SRC		NRAS	
			PDGFRA	
			PDGFRB	
			RAF1	

*Negative Effects*				
TP53	PIK3C1/AKT	PI3K2	RAS	RB
MDM2	PTEN		CBL	CCND1
MDM4			ERRFI1	CCND2
			SPRY2	CCNE1
				CDK2
				CDK4
				CDK6

We split all genes into those with negative effects on the pathway and those with positive effects. Each column is headed by the name of the signaling pathway in which the genes reside, followed by the list of genes. A gene with positive effect increases the generation of the pathway's product when amplified, while a negative effect gene decreases its production when amplified. Deletion of a gene has the reverse effect on the pathway.

**Figure 2:**
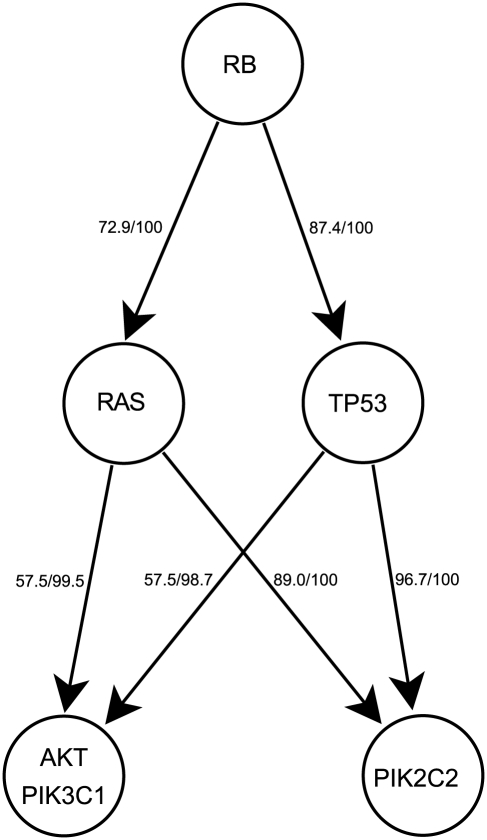
Temporal sequence of pathway alterations in all samples. Alterations included as alterations of each pathway are defined in Table 1. Each arrow indicates the order in which the two alterations arise. The first number represents the frequency with which the displayed temporal sequence occurs. The second number represents the percent of all bootstrap iterations in which the order determined acts as the dominant temporal sequence.

We then applied the RESIC algorithm to pairs of co-occurring pathway alterations and determined a general order for signaling pathway disruptions. When using all GBM samples in a single computational analysis, we found that RB signaling alteration occurs early during gliomagenesis. In contrast, AKT and PI3K2 signaling alterations occur late, while RAS and TP53 signaling changes arise in between the early and late events (Figure 2). As observed in the CNA-only versus CNA with point mutation validation analysis, the addition of point mutation information leads to earlier placement of point mutation-driven alterations. Thus, TP53 pathway alteration may actually arise earlier, given that *TP53* alteration tends to be due to point mutation. In no case were these signaling pathway alterations driven primarily by a single mutation. While the placement of RAS pathway alterations before AKT/PIK3C1 pathway changes seems to be in contrast to our previous results involving EGFR (part of the RAS pathway) and PTEN (included in the AKT/PIK3C1 pathway) [24], in actuality, the earlier result does not preclude the possibility that other mutations in the RAS pathway arise even earlier. The benefit of the pathway-driven approach is that these sporadic mutations can be combined into a single event for a better comparison of functional consequences such as (in)activation of pathway signaling. For the detailed output of the orderings determined by RESIC for the GBM dataset, see Text S1.

While biological investigations of primary GBMs have not determined the order in which pathways are altered, the possibility arises to compare our results to secondary GBMs, for which the order of events can be inferred from the tumor grade. We thus compared the results of our RESIC pathway analysis against literature evidence about the order of events in secondary glioblastomas. Disruption of RB signaling as an early event is consistent with both the necessity of passing the G1/S cell cycle checkpoint to allow the development of a tumor and the observation of RB pathway mutations in the majority of low- and high-grade gliomas [43]. While TP53 loss-of-function point mutations occur in most low-grade gliomas [44], [45], our restriction to solely CNAs excludes such mutations from the analysis. Despite not including TP53 point mutations in the analysis, we obtained the results that TP53 pathway alteration occurs early during tumorigenesis via MDM2 and MDM4 alterations. Inclusion of point mutations would only increase the frequency of TP53 pathway alterations and thus place TP53 pathway events even earlier, as seen in our CNA data-only validation analyses. In vitro studies confirm the positioning of the remaining pathway alterations: previous results in cell lines showed that AKT signaling alteration contributes to the progression of anaplastic astrocytomas to GBMs [46], but cannot initiate tumorigenesis without RAS activation [47]. Additionally, previous work showed that RB alteration, TP53 inactivation and hTERT expression in combination with RAS activation resulted in the malignant transformation of normal cells; however, the emergence of PI3 kinase alterations with RB, TP53, and hTERT changes without RAS alterations were not capable of inducing similar responses [48], thereby placing both AKT/PIK3C1 and PI3KC2 pathway alterations relatively late during malignant transformation. These in vitro studies in secondary GBMs provide independent support for our results in primary GBMs.

### RESIC identifies distinct temporal sequences of pathway alterations for GBM subtypes

**Figure 3:**
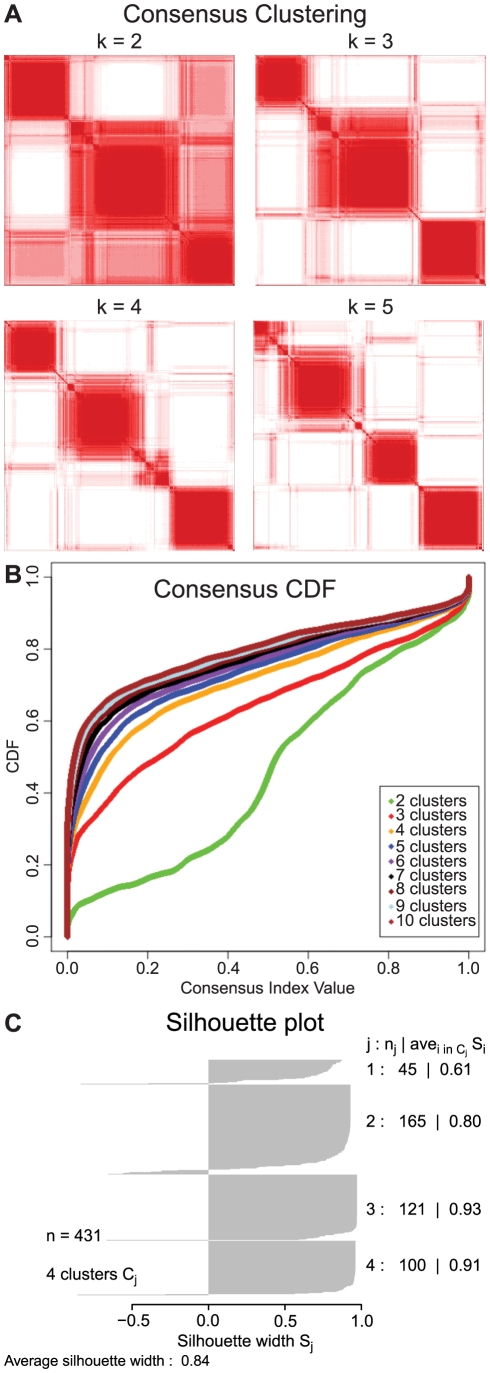
Cluster analysis of the TCGA GBM patient data. The stability of the clusters increases with the number of clusters and stabilizes around four clusters. **A**) Consensus matrix. The entry (*i, j*) in the consensus matrix measures the proportion of iterations of clustering in which the *i*th sample clusters with the *j*th sample. Assuming perfect clustering, all entries (*i, j*) in the consensus matrix would be either 0 or 1, representing either sample *i* never clustering with sample *j*, or always clustering with sample *j*. When the samples in the matrix are ordered according to their cluster, perfect consensus results in a block-diagonal matrix. Note that the stability of the clusters stabilizes at *k* = 4. **B**) Consensus CDF of the entries of the consensus matrix. With perfect clustering, all entries would be zero or one, resulting in a CDF consisting of a flat line at the percentage of zero entries in the consensus matrix and ending with a spike at one. The closer the CDF approaches this limit, the better the clustering. Note that the clustering stabilizes at *k* = 4, with little increase afterwards. **C**) Silhouette plot of the four clusters. Positive values on the silhouette plot identify samples that most stably represent each subtype. We exclude samples with zero or negative silhouette values to ensure only samples that fit the subtype are used in the subtyped RESIC analyses.

Recent findings demonstrate the existence of distinct molecular subtypes of GBMs [33], [34], [35], and we hypothesized that the order in which pathways alterations arise may vary between subtypes. We used gene expression profiling following the approach of Verhaak et al. [35] to cluster GBM samples into the previously described subtypes. In brief, we used Consensus Clustering, a form of hierarchical clustering with agglomerative average linkage, to determine the set of clusters in the gene expression data (Figure 3A and B) [49]. Positive silhouette widths were then used to identify the subset of samples that fit well into each subtype (Figure 3C) [50]. Thus, we obtained 98 samples of the classical subtype, 157 of the mesenchymal, 43 of the neural, and 120 of the proneural subtype (see Methods for details). These frequencies of samples in each subtype were similar to the frequencies determined in previous studies [5], [34], [35], [51], [52]. Additionally, since some previous subtyping efforts were done utilizing TCGA data, we compared these results to ours and found a very high concordance (>90%) in the subtyping of the shared samples [35]. These results implied that the clusters we determined corresponded to the subtypes previously determined. By applying our pathway-based RESIC approach to each of these clustered sets of samples individually, we then tested whether the temporal order of pathway alterations detected at the subtype level corresponded to the same or similar order of events as detected earlier when all samples were analyzed. Each subtype-specific RESIC analysis was performed in the same manner as the overall pathway-based GBM analysis (see Methods). We detected a significant temporal order for the two largest subtypes (proneural and mesenchymal GBM), some correlations but no significant order in the second smallest type (classical GBM), and no correlations in the smallest subtype (neural GBM). We found distinct differences between the subtypes with significant temporal sequences, as outlined in the following.

### Proneural GBM

**Figure 4:**
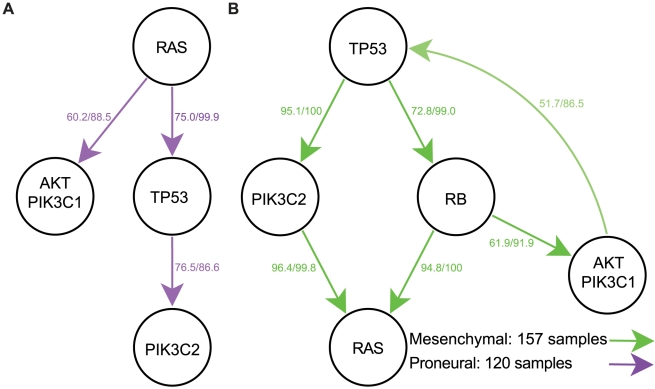
Temporal sequence of pathway alterations within subtypes. The classical and neural subtypes did not result in any significant orderings of pathway alterations. Mutations included as alterations of each pathway are defined in Table 1. Each arrow indicates the order in which the two alterations arise. The first number represents the frequency with which the displayed temporal sequence occurs. The second number represents the percent of all bootstrap iterations in which the order determined acts as the dominant temporal sequence. **A**) The proneural subtype. **B**) The mesenchymal subtype.

The second largest cluster of samples was the proneural subtype with 120 samples. This subtype is characterized by PDGF-related amplifications and TP53 point mutations [35]. We found that the temporal sequence of pathway alterations in this subtype closely recapitulated the overall pathway order results. The primary difference between the two was a lack of a significant ordering involving the RB pathway in this subtype. Additionally, RAS pathway alteration was placed before TP53 pathway inactivation. Interestingly, we detected a divergence in the temporal order between PIK3C1/AKT (AKT1/2/3, GAB1, IRS1, PIK3CA/B/D/G, PIK3R1/2, PDPK1, SRC, PTEN) and PIK3C2 (PIK3C2A/B/G) signaling (Figure 4A) even though both pathways involve the lipid-kinase activity of PI3K family enzymes [53]. In particular, we found that PIK3C2 pathway alterations require subsequent TP53 pathway inactivation, through alteration of MDM2/4, TTP53, and/or CDKN2A, while PIK3C1/AKT alterations do not (Figure 4A). This difference may be explained through the downstream effects of PIK3C1 signaling: specifically, in the PIK3 class 1 pathway, PIK3 class 1 family proteins produce PIP3, which results in downstream activation of AKT and activation of the MDM2 gene; the latter then inactivates TP53 signaling [53]. By contrast, PIK3 class 2 enzymes catalyze formation of PI-3-P, which does not activate AKT to inactivate TP53 signaling [53]. As such, the set of samples with AKT/PI3KC1 signaling activation may not require TP53 signaling inactivation while for the set of samples with PIK3C2 activation, inactivation of the TP53 network may be necessary. Thus, the two divergent temporal sequences may represent a difference in the biology of PIK3 class 1 mutated versus class 2 mutated patients in the proneural subtype. For the detailed output of the orders determined by RESIC for the proneural subtype, see Text S2.

### Mesenchymal GBM

The largest GBM subtype was the mesenchymal subtype with 143 samples. Even though this subtype is characterized by NF1 alterations [35], our GISTIC analyses did not detect any significant copy number alterations of the NF1 locus. Indeed, loss of the NF1 protein can be driven by multiple mechanisms including heterozygous copy number changes, inactivating point mutations, and low levels of gene expression without genomic alterations [3]. We therefore excluded NF1 from our analysis and focused on the remaining copy number changes. Unlike the proneural subtype and in contrast to the overall results derived from all samples, RESIC predicted that samples of the mesenchymal subtype first accumulate TP53 pathway inactivation, followed by PIK3C2 and RB pathway alterations, and concluding with RAS and PIK3C1/AKT changes (Figure 4B). One possibility for the early placement of TP53 pathway inactivation is that in this subtype, the TP53 pathway is frequently altered via MDM2, MDM4, or CDKN2A CNA events, leading to an increased number of samples with CNA in the TP53 pathway and thus an early placement of that pathway. The lack of an inferred order between the RAS and PIK3C1/AKT pathways may potentially be the result of increased crosstalk between the two pathways in this subtype: cell line experiments have shown that while both RAS and PIK3C1/AKT pathway alteration may be necessary to initiate cancers, either pathway alone is sufficient to maintain tumor growth [54]. In addition, we observed that in a subset of cases, AKT alteration was predicted to occur after RB signaling disruption and before TP53 inactivation, representing the reverse order of the overall temporal sequence. This finding may be due to the accumulation of multiple mutational hits at different times in one or more of the pathways, or due to feedback between the gene products in the pathways. Given that the reversed ordering had less weight than the overall order, a second hit in a subset of samples could also potentially account for this reversed order. For the detailed output of the orders determined by RESIC for the mesenchymal subtype, see Text S3.

### Neural and classical GBM

For samples belonging to the neural and classical subtypes of GBM, no significant temporal sequence of pathway alterations was determined. There were only 43 samples classified as neural GBM, which is a limited number of samples on which to perform RESIC analyses. In addition, the neural subtype is often subject to contamination, a finding supported by the observation that normal tissue samples cluster together with samples of this subtype [35]. We then analyzed the 98 samples belonging to the classical GBM subtype. Huse et al. recently found that all samples categorized as classical GBM according to Verhaak et al. [35] can instead be split evenly into the remaining three subtypes [55] when following the previous classification scheme by Philips et al. [34]. Thus, for this subtype, we expected to detect an even distribution of temporal orderings corresponding to the other subtypes. Indeed, we found no significant temporal orderings of events, but an even distribution of the flow through the mutational network (see Text S4 and S5).

### RESIC identifies distinct temporal sequences of genetic alterations for GBM

**Figure 5:**
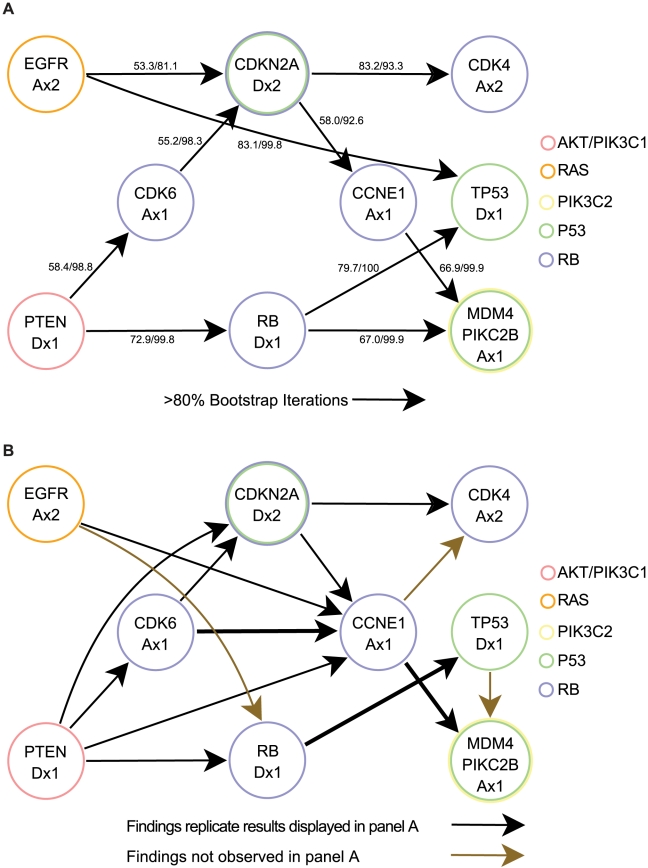
Temporal sequence of somatic mutations in all samples. Each arrow indicates the order in which the two alterations arise. **A**) Map of the temporal order of all CNAs determined using pairwise RESIC analyses. The first number represents the frequency with which the displayed temporal sequence occurs. The second number represents the percent of all bootstrap iterations in which the order determined acts as the dominant temporal sequence. **B**) Map of all CNAs made using three-mutation RESIC analyses. We tested the effects of including additional mutations in RESIC analyses by first testing the addition of a single mutation independently to each analysis. Investigation of further additions of mutations would require more samples; furthermore, we would expect any epistatic effects on the order of mutations to show some level of effect from each gene independently. Arrows in black are significant orderings, confirmed in at least 80% of the bootstrap iterations. Gold arrows are orderings found significant by three-way interactions, but not by pairwise interactions. Thickness of lines denotes the number of interactions that maintained the ordering. Since multiple three-gene analyses correspond to some arrows, the specific frequencies of orderings and the number of bootstrap iterations are not displayed, although included in the Supplementary Information. The results of using two mutations per RESIC analysis (A) do not differ significantly from the three mutation results (B). In no case is an order determined to be significant in pairwise analyses later found to be reversed in three way analyses. Additionally, we found that most results are stable (confirmed in three-way analyses) as long as the most likely evolutionary path through the mutational network comprises at least 58% of the flow. With the exception of the placement of PTEN of the AKT/PIK3C1 pathway, many of the orderings determined at the pathway level are robust at the gene level.

After determining the order of pathway alterations both in all GBM samples and in specific subtypes, we sought to establish the temporal sequence of specific genetic alterations underlying these pathway alterations. We identified all correlated alterations within the TCGA dataset with a p-value of 0.05 and analyzed the results using the RESIC algorithm. We first analyzed the alterations on a pairwise basis: if two alterations were correlated, we performed a RESIC analysis on the pair and determined the relative order of the two alterations. We then combined all relative orderings into a single network (see Methods). In these analyses of genetic alterations, there was no instance in which one alteration was placed early by one analysis but late by another analysis, demonstrating consistency between the results despite the assumption of independence between individual analyses (Figure 5A). Additionally, our results involving EGFR and PTEN correspond closely to the findings obtained previously [24]: we found a loss of significance of the ordering when only CNA data was included, resulting in frequencies of 43.5% versus 46.3% for the flux with PTEN before EGFR and low level amplification of EGFR before PTEN loss, respectively, which is consistent with our current results of 43.8% versus 45.9%. We did, however, obtain a result that was not consistent with the progression of events inferred from secondary glioblastomas: PTEN loss was placed early in our analyses while in secondary GBMs, PTEN loss tends to occur late, rarely occurring in astrocytomas; in contrast, PTEN alterations are prevalent in GBMs [56]. This result may be due to differences in progression between primary and secondary gliomas.

Additionally, in contrast to our pathway-driven analyses, we found that many of the resulting orderings were weaker when using individual gene-based analyses (Figures 2 and 5). For example, in pathway-based analyses (Figure 2), four out of the six temporal orderings were determined to occur with more than 70% frequency as well as in 100% of all bootstrap iterations. In contrast, in gene-based analyses (Figure 5), only four of ten temporal orderings occurred with more than 70% frequencies and in less than 100% bootstrap iterations. This difference exposes the additional level of noise that gene-based approaches encounter due to the similarity of the effects of mutations within genes belonging to the same pathway. With the exception of the placement of PTEN in the AKT/PIK3C1 pathway, many of the orderings determined at the pathway level were the same at the gene level. However, in gene-based analyses, the true orderings can be obscured by the fact that multiple genes are involved in each pathway. Other consistencies between pathway- and gene-level analysis include RB pathway alteration through RB deletion occurring before P53 pathway inactivation through MDM4 and before PIK3C2 pathway inactivation through PIKC2B, RAS as an earlier event through EGFR amplification, and TP53 mutation arising before PIKC2B (Figure 5).

We then aimed at investigating the robustness of the RESIC results to inclusion of further genomic alterations into each individual analysis. Addition of further alterations may perturb the ordering determined in the pairwise RESIC analysis by providing an alternative set of paths through the network. That is, while the total numbers of patient samples harboring unmutated cells, low-level alteration, and high-level alterations remain the same, these numbers are distributed among a larger number of mutational states due to the inclusion of further alterations in the network. If a significant fraction of the flux through the network is then diverted to one particular mutational state, then the predicted order of events might change. In principle, to determine the most accurate sequence of events in cancer progression, all alterations involved in tumor development would need to be included in a single RESIC analysis. However, such an analysis is infeasible: combinatorial expansion of the number of mutational states precludes the existence of a sufficiently large number of samples to populate a significant fraction of the mutational states. Instead, to test the effects of including additional alterations on the obtained RESIC results, we performed three-way RESIC analyses on the full TCGA data set. We thereby independently tested the effect of including every additional alteration correlated with the pair of alterations investigated in the pair-wise analyses. We found that in all cases in which a single temporal sequence of events arises at least 58% of the time at the pair-wise gene level, the results were stable between pair-wise and three-way analyses and thus unaffected by the addition of further alterations to the analysis (Figure 5B). In some cases, notably for networks of EGFR and TP53 and networks of RB and MDM4/PIK3C2B, the order of events remained constant between pairwise and three-mutation analyses; however, an additional alteration was placed in between these changes. Thus, an additional temporal ordering was determined by using three-mutation analyses (Figure 5B).

### Subtype-specific RESIC analyses of somatic alterations

**Figure 6:**
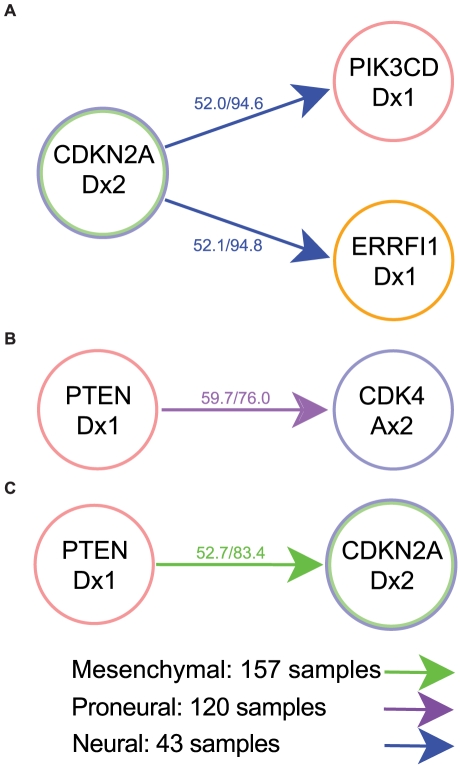
Temporal sequence of somatic mutations within subtypes. The classical subtype did not result in any significant orderings of genetic alterations. **A**) Neural subtype. **B**) Proneural subtype. **C**) Mesenchymal subtype. In contrast to the results obtained when using pathway alterations, we obtained no significant results when performing gene-level analyses. In most cases, the temporal sequences occur with less than 58% probability, meaning the results are unlikely to remain robust to perturbation by addition of further genes. In the neural subtype, the sole significant temporal order determined involves PTEN loss arising before CDK4 amplification.

Since we detected differences between the GBM subtypes when analyzing the temporal order of alterations of signaling pathways, we expected those differences to also appear at the genetic level. Thus, we analyzed the subtype-specific sequence of somatic genomic alterations. Due to the small sample numbers, the temporal order of alterations within subtypes could be determined in only a few cases (Figure 6). Consistent with results obtained using the pathway-based approach, we obtained no significant temporal orderings for samples belonging to the classical subtype. By contrast, for samples of the neural subtype, we obtained significant orderings of somatic alterations (Figure 6A). However, the frequency of the dominant temporal sequence for those cases was only slightly greater than 50%, suggesting that these findings were not robust. Indeed, the use of Bonferroni corrected p-values for correlations removed the significance of these results, as did the use of three-way analyses. For samples belonging to the proneural and mesenchymal subtypes, we detected only one significant ordering each, both involving PTEN deletion events (Figure 6B and C). The order determined for PTEN and CDKN2A in the mesenchymal subtype, like the ordering found for the neural subtype, had a flux through the network of about 50% and a p-value for correlation of 0.05, and therefore was not significant. The order of PTEN and CDK4 determined for samples belonging to the proneural subtype, however, passed the threshold of 58% flux through the network and also had a p-value for correlation of 0.02, and was therefore more likely to be significant. The lack of significance in the analysis of the subtypes with respect to genetic versus pathway alterations illustrates the increased ability of the pathway-driven approach to detect the temporal sequence of events with fewer samples.

In summary, the use of pathway information for the RESIC analyses clarified the order of events in several ways. First, combining mutational events into pathways allowed for the detection of an ordering in cases in which fewer samples and potentially conflicting genetic signals obscured any significant ordering of events at the genetic level (Figures 4 and 6). In cases in which an order could be determined at the genetic level, the increased number of events often resulted in an additional level of noise, thereby obscuring the results. In addition, using pathway information removes some potentially incorrect orderings through merging of specific alterations with the remaining events in the pathway. However, the use of pathway information could also hide clinically relevant mutational anomalies that should arise separately from alterations of the remainder of the pathway. In our GBM analyses, the placement of PTEN loss is such a concern.

## Discussion

In this paper, we have extended the RESIC methodology [24], originally designed to identify the temporal sequence of somatic genetic alterations from cross-sectional tumor samples, to address the order in which modifications of molecular signaling pathways arise during tumorigenesis. This modification considerably reduced the noise compared to the use of individual genetic alterations. Furthermore, it makes RESIC applicable to tumor types in which individual mutations may be so infrequent that they rarely co-occur in samples, thus leading to the absence of statistically significant associations among events. In such cases, a pathway-based application of RESIC groups together these sporadic events and allows for the analysis of the temporal sequence of events.

We have applied our methodology to primary glioblastomas (GBMs) and have identified the temporal ordering of both pathway-level and gene-level alterations. These investigations were performed on the entire group of glioma samples and each of four previously defined molecular subtypes of GBMs [35]. The subtypes are characterized by unique RNA expression and mutational patterns [55] and, interestingly, differed in their chronologic acquisition of molecular events. The order of events was clearest for the proneural and mesenchymal subgroups. This observation was consistent with data suggesting that these two groups are more distinct and reproducible between large expression analyses of GBM tumor collections. Moreover, within the proneural group, the existence of two distinct mutational pathways is interesting given the emerging data that this subgroup may indeed be a mixture of at least two distinguishable molecular subtypes [55]. It is conceivable that these differences may be explained by differences in the cell of origin of each subtype; further work is required to determine whether these two paths of mutational acquisition are related to the disparate clinical behavior of this GBM subgroup, although the outcome for GBM is quite poor in almost all cases [57].

In summary, our method allows the rational investigation of genetic and pathway alterations arising during tumorigenesis and will be an important tool for the field to determine the order of acquisition of events from cross-sectional cancer databases.

## Methods

### Patients and tumor samples

We obtained copy number alteration (CNA), gene expression, and sequencing data for all GBM samples for which CNA data were available from the TCGA Data Portal at http://tcga-data.nci.nih.gov. These samples were collected and processed by the TCGA Biospecimens Core Resource [3]. We ensured that each sample represented a unique case by excluding all patients who were represented in the database with multiple samples that had different copy number results for each sample. We thus obtained 462 samples. All three data types were obtained in TCGA Level 3 format. For gene expression data, this means that all data had been previously log-transformed and median centered, while CNA data had been processed from microarray fluorescence amplitudes into segmented data files. Sequencing data had been called from amplitude data as specific indels or point mutations [3]. Descriptions of the TCGA levels can be found at http://tcga-data.nci.nih.gov/tcga/tcgaDataType.jsp.

### Detection of copy number alterations (CNAs)

In order to determine significant CNAs, we applied the GISTIC algorithm [5] to the segmented TCGA GBM copy number data. Independent runs of the algorithm were performed on the complete set of samples as well as samples belonging to individual subtypes. These analyses were run using the GenePattern analysis software [58].

### Determination of the CNA rate

We considered the accumulation of focal CNAs. Unlike whole chromosome or chromosome arm gains or losses, these CNAs are not caused by gross karyotypic abnormalities, likely due to chromosomal instability [36]. Indeed, selective pressure and the fitness effects of mutations can drive their accumulation without the need of elevated mutation rates [36], [59], [60], [61]. We calculated the rate of focal copy number alterations based on a per generation copy number alteration rate of 4.2×10^−5^ alterations per locus in sperm [62], [63]. As previously determined, the number of cell divisions, *N_x_*, that sperm germ cells undergo is determined by the following formula: *N_x_* = 30+23 (*x*−*x_p_*)+6 = 36+23 (*x*−*x_p_*), where *x* is the age of the male and *x_p_* is the age at puberty [64], [65]. Then, with an average generation of twenty years and an average age of puberty of 13, the number of cell divisions in spermatogenesis is on average 191. Dividing the per generation rate by the number of cell divisions per generation, we determined the per cell division per locus rate of CNAs as 2.2×10^−7^, or 1.1×10^−7^ per allele. Coincidentally, this rate corresponds well with the mutation rate we previously used for point mutations per allele per cell division [24], and agrees with evidence that point mutation and focal copy number alteration rates must be similar in scale such that both mutation types are observed in similar frequency [36]. We simplified and used μ = 1.0×10^−7^ mutations per allele per cell. In contrast, whole chromosome or chromosome arm gains or losses are likely to arise through chromosomal instability, and may occur at a much higher rate [36].

### Gene expression-based subtyping

We used the gene expression profiling to cluster the samples into the four subtypes of GBM using Consensus Clustering [49] following the approach of Verhaak et al. [35]. We used the 1,740 genes in Verhaak's gene signature as input to the Consensus Clustering algorithm [35]. We applied hierarchical clustering with agglomerative average linkage as the basis for Consensus Clustering, with a distance measure of 1 minus the Pearson correlation coefficient, and 1,000 iterations with a sub-sampling ratio of 0.8. As the Consensus Clustering algorithm requires as input the number of clusters, *k*, in which to place the samples, we chose 4 clusters [33], [34], [35], but tested *k* = 2 to 10 clusters. We observed increasing stability of clusters via graphical examination for increasing *k* until *k* = 4 clusters (Figure 3A and B), but no significant gain in stability afterwards. Once the clusters were determined, we used the silhouette method to restrict the samples used to only those with positive silhouette widths (Figure 3C). Silhouette width is defined as the ratio of each sample's average distance to all other samples in the same cluster and the average distance to all other samples [50]. Thus, those samples with positive widths are more closely related to the samples within the cluster than the samples outside the cluster, and are likely to reside in the correct cluster. The R package Silhouette was used to determine silhouette widths [66].

### Determination of pathway events

Mutations in several different genes can result in phenotypically similar results. Indeed, network analyses of TCGA GBM samples have detected a tendency for alterations to occur in specific functional modules instead of particular genes [31]. Investigators of the TCGA project defined a set of genes commonly mutated in TCGA glioblastoma samples and mapped them into commonly altered GBM signaling pathways [3], [31], [32], visually depicted in Supplementary Figures 7 and 8 of [3]. Specifically, they mapped the CNAs and point mutations commonly found in the TCGA GBM dataset onto a manually generated network diagram of glioma genetics gleaned from Furnari et al. [67]. We opted to use this classification of pathways for several reasons: first, the set of pathways was designed using the TCGA dataset and is specific to GBMs; it thus provides greatest specificity and applicability to the sample set we investigated. Second, the pathways were manually curated from the glioma literature [67], ensuring as much accuracy in the pathways as currently possible. Finally, the directionality of the effect of each mutation could be inferred from this definition of pathways. Manually curated pathways are not the only option: pathway software such as Ingenuity IPA (http://www.ingenuity.com) or BioPax [68] can identify the common pathways in a given dataset based on databases of known gene or protein interactions. However, these pathways would not be as specific to GBM data, especially TCGA data, as the pathway definition used by the TCGA consortium itself. The pathways may also harbor inaccuracies due to limitations of software parsing of interactions. Finally, computer-generated pathway definitions tend to lack the directionality of the effects of mutations. Knowledge of whether a particular mutation will increase rather than decrease signaling in a pathway simplifies the analysis.

Using the TCGA classification as a first level approximation for sets of genes with similar phenotypic effects, we split genes according to those signaling pathways (Table 1) [31], [32]. We then determined whether a specific alteration in a gene would result in an increase or decrease in signaling downstream of the gene. While in this particular pathway definition, the directionality of effect of mutations is known, it is possible to perform a similar analysis without such information. Should the pathway information available not include whether a given mutation increases or decreases pathway signaling, as is the case with many pathway databases like Ingenuity or Biopax, we assume that the more frequent alterations of any given gene (e.g., amplification or deletion) result in a pro-tumorigenic effect on cells, as functional mutations detrimental to cancer progression are unlikely to occur frequently. However, inferring network and pathway information may introduce further errors, as pathway events may be wrongly defined. Thus, if non-directional pathway information is used, caution is needed to ensure maximum accuracy.

We considered a single alteration event occurring within any of the genes in the network to be sufficient to result in a change in signaling of that pathway. Since only focal copy number alterations were considered, and since in most cases, alterations within a signaling pathway reside on separate arms of different chromosomes, we considered the first mutational hit on each gene to be independent of all other mutations in the pathway. Thus, a particular pathway alteration had a mutation rate equivalent to *Mμ*, where *M* is the number of genes involved in the pathway and *μ* is the per gene mutation rate. In a similar manner, the TCGA copy number alteration data [3] was transformed into pathway alteration data by considering a positive alteration status as having one or more mutations that increased pathway signaling, a negative alteration status as having one or more mutations that decreased signaling down the pathway, and a neutral status when no mutations within the pathway occurred.

### Selection of biologically relevant genomic alterations

We restricted the set of genes related to GBM development to those that were defined in the pathway diagram in [31], [32]. The list of genes considered for analysis is provided in Table 1.

### Calculation of the pairwise temporal order of events through RESIC analyses

We used the RESIC methodology [24] for the calculation of the order of genomic events in cancer. RESIC considers an initial population of *N* cells at risk of accumulating the genetic changes leading to cancer. These cells proliferate according to the Moran process [25], a stochastic process in which a cell is chosen in proportion to its fitness to divide. Each division results in two daughter cells, one of which replaces the original cell and the other replaces a randomly chosen cell. During each division, one of the daughter cells may accumulate a mutation. The lineage of a mutated cell can then take over the population (i.e. reach fixation) or go extinct due to stochastic fluctuations. This Markov process is used to describe the dynamics of mutation accumulation in a population of cells, for each patient.

Depending on the fitness values of the various combinations of potential mutations, each path through the network of mutations may have a different likelihood of occurring. The Markov process described above can then be used to calculate the transition rates between individual mutational states, and thus the likelihood of each path through the network. This model is then used to describe the dynamics of populations of patients, each harboring a population of cells at risk of accumulating mutations. The scaling from this micro-model (on the level of a population of cells within a single patient) to the macro-model (on the level of a population of patients, each with a population of cells proliferating according to this stochastic process) is obtained by multiplying the transition rates between mutational states within one patient by the number of patients in each mutational state. We considered the dynamics of patients in steady state: there is a constant influx into the unmutated state and an equal constant outflux from the fully mutated state, accounting for diagnosis of the disease (influx) and deaths of patients or their cure (outflux). These parameters are selected to ensure that fitness values are within biologically meaningful ranges, ie. that a single mutation does not confer too large a fitness effect to be biologically implausible. According to this model, patients enter the system in an unmutated state and then accumulate the mutations of interest. This starting point could be late during tumorigenesis, if the mutations of interest are late events, or pre-cancerous if the mutations of interest are initiating events; either way, since the predictions of the model are compared to data of patients after diagnosis of their disease, this starting point in the model is also after diagnosis of the disease. Note that small influx values into other mutational states, representing diagnosis with a different set of alterations, does not significantly alter the results.

At steady state, the population is distributed across all possible states; this steady state distribution can then be compared to the numbers of clinical samples with the corresponding genotypes, where the total number of patients in a dataset is equal to the sum of patients in all states. Since all samples analyzed are collected from patients after diagnosis of their disease, the earliest time point identifiable using such datasets is the time of diagnosis. As outlined above, this definition does not imply that the first mutation arose exactly at the time of diagnosis: since the event resulting in patients entering the system is diagnosis, and all considered events are observable in the patient samples, many events arise prior to diagnosis. This mapping is used to optimize a subset of parameters in the Markov process model (i.e. the fitness values of cell types) by minimizing the difference between the prediction and the observed frequencies in the dataset. Other parameters, such as cellular population size, mutation rate and influx rate, are estimated from experimental results [36], [69] and tested for robustness over several orders of magnitude [24]. The output of RESIC is given as percent of the flux through the network via each particular path. We found previously that within multiple orders of magnitude, changes in the population size, mutation rates, and in- and out-flux values have little influence on either the relative order of the fitness values determined for each combination of mutations, or the final percent flux through each evolutionary trajectory.

Similarly, Beerenwinkel et al. found that the selective advantage of mutations has the largest effect on the evolutionary dynamics of tumorigenesis [70]. While their model was designed using the Wright-Fisher stochastic process rather than the Moran process in our model, both of our approaches found that changes in population size and mutation rate alter the absolute fitness values necessary to result in the same trajectory towards cancer [24], [70]. Thus, there is a dependence on the dynamics of the system onto parameters such as the fitness values of cells harboring particular combinations of alterations. In our model, these changes in fitness values, however, are so small that they still result in similar results for the temporal sequence of mutations [24].

### Determination of independent temporal sequences of events

We applied the RESIC methodology [24] to the set of events of interest (gene-based or pathway-based, subtyped samples or total sample set). We determined the level of correlation between all altered pathways using Fisher's Exact Test. Positive correlation is required to ensure that events co-occur sufficiently frequently to allow for a determination of the temporal sequence. Applying a p-value cutoff of 0.05, we identified all significantly correlated pathway alterations and performed RESIC analyses on each pair of correlated altered pathways. We used the following set of parameters: the mutation rate was set to μ = 2.0×10^−7^ per gene in the pathway per cell division and the stem cell population size per niche was set as *N* = 100. This choice of value for *N* was made for computational speed; we had previously found that an order of magnitude variation in this parameter value results in robust results [24]. As the RESIC algorithm is stable to order of magnitude changes in the mutation rate as well [24], these parameter choices led to robust results. A particular order of alterations was considered significant if at least 80% of all bootstrap iterations resulted in the same dominant sequence. The bootstrapping procedure was performed independently for each analysis. In each analysis, we took our original data set of N patients and then obtained a bootstrap sample of size N by using sampling with replacement from the counts of patients with each potential combination of mutation. We performed 10,000 bootstrap iterations per analysis. We then applied the RESIC algorithm on the bootstrap sample to determine the temporal sequence of events given the bootstrapped patient population. The bootstrap percentage for a given order of events is the percent of all bootstrap iterations in which a given order of events is the most likely order of events. The frequency of a given order of events displayed in our figures is the average frequency over all bootstrap iterations.

### Validation of robustness of the results to exclusion of point mutation rate

We applied the RESIC methodology to the 145 TCGA patient samples for which CNA and point mutation data was available. We analyzed two sets of data: the dataset including CNA only, and the dataset containing CNA and point mutation date. We analyzed both datasets through our pathway-based approach as well as the gene-based approach using pairwise and three-way analyses. We compared the resulting temporal orderings, considering the order to be consistent between the two data types when the percent flux through the mutational diagram varied by no more than three and the dominant temporal sequence remained dominant. We considered an ordering to be weakened if either fewer alleles of a gene could be analyzed, the percent flux decreased by more than three, or a pair or triplet of events could no longer be analyzed when using CNA data only. We considered an ordering to be inconsistent if the dominant temporal order changed between the two analyses, or if an ordering could be determined when using CNA data only, but not when using CNA and point mutation data.

### Combining individual RESIC analyses to determine the overall sequence of events

In pair-wise analyses, the temporal order of all alterations can be determined simply by combining the pairs of genetic or pathway alterations in the predicted order. For example, if mutation A arises before mutation B, and mutation C arises before mutation A, the order of events is C→A→B. Figure S1 depicts all possible combinations of pairs of mutations that result in a three-mutation ordering. Unlike pair-wise analyses, three- or more-way analyses cannot be done simply by combining the sets of genes involved, since there may be overlap between the results. Instead, we built upon the pair-wise results we had determined first and then overlaid the three-way results. Pair-wise orderings of genes that were confirmed in multiple three-way analyses represent stable temporal sequences of events. The resulting temporal ordering of all mutations, with thicker lines for pairs of genes for which multiple analyses confirmed the same order, is shown in Figure 6B.

## Supporting Information

Figure S1 Pairwise comparison of three genes results in an ordering of all three genes. We perform pairwise comparisons between alterations to determine the overall order of events. Here we depict all possible combinations of two pairwise orderings that can result in a single linear order of events. We denote the three events A (in red), B (in blue), and C (in green). Black arrows denote the determined orders and gray arrows denote the set of pairwise orderings that result in a three-event order.
(EPS)

Text S1 RESIC analysis results for all GBM samples. The results from the pairwise, three-way, and pathway-based RESIC analyses are displayed.
(TXT)

Text S2 RESIC analysis results for GBM samples belonging to the proneural subtype. The results from the pairwise, three-way, and pathway-based RESIC analyses are shown.
(TXT)

Text S3 RESIC analysis results for GBM samples belonging to the mesenchymal subtype. The results from the pairwise, three-way, and pathway-based RESIC analyses are displayed.
(TXT)

Text S4 RESIC analysis results for GBM samples belonging to the neural subtype. The results from the pairwise, three-way, and pathway-based RESIC analyses are shown.
(TXT)

Text S5 RESIC analysis results for GBM samples belonging to the classical subtype. The results from the pairwise, three-way, and pathway-based RESIC analyses are shown.
(TXT)
